# The complete chloroplast genome sequence of *Citrus maxima* (Burm.) Merr. ‘Guanximiyou’

**DOI:** 10.1080/23802359.2019.1704661

**Published:** 2020-01-10

**Authors:** Shi-Rong Xu, Chang-Yue Huang, Ya-Ting Deng, Lin-Li Zhou, Dong-Ming Pan, He-Li Pan

**Affiliations:** College of Horticulture, Fujian Agriculture and Forestry University, Fuzhou, PR China

**Keywords:** Chloroplast, *Citrus maxima*, ‘Guanximiyou’

## Abstract

*Citrus maxima* (Burm.) Merr. ‘Guanximiyou’ is a major citrus tree largely cultivated in China. A previous study has reported the complete chloroplast genome of *C. maxima*, but there may be some differences between wild species and cultivating variety. In this study, the complete chloroplast genome sequence of ‘Guanximiyou’ pummelo was characterized using BGISEQ-500 sequencing. The chloroplast genome was 160,186 bp in length and separated into four distinct regions such as large single-copy region (87,939 bp), small single-copy region (18,395 bp), and a pair of inverted repeat regions (26,926 bp). The genome contained a total of 109 genes including 79 protein-coding genes, 29 tRNA genes, and 4 rRNA genes. Phylogenetic maximum-likelihood analysis revealed that ‘Guanximiyou’ pummelo was clustered with other Rutaceae species with high bootstrap values.

‘Guanximiyou’ pummelo, belonging to the subfamily Aurantioidae of the Rutaceae, is an important agricultural product with immense economic value (Li et al. 2015). At present, ‘Guanximiyou’ pummelo is one of widely cultivated in Fujian, Sichuan, Zhejiang, Guangxi, Hainan, and some other southern provinces of China, owing to its unique taste, fragrance, and seedless. In addition, there are 11 bud mutations originated from ‘Guanximiyou’ pummelo, showing different harvesting date and various colors in flesh or peel, which greatly enriches citrus varieties and is an important material for studying the mechanism of bud sport. In this study, we characterized the complete chloroplast (cp) genome sequence of ‘Guanximiyou’ pummelo and explored its phylogenetic relationship with several citrus species, which would provide valuable basic genetic resource for the plants of Rutaceae.

The fresh leaves sampled from a mature ‘Guanximiyou’ pummelo tree, which was growing in a citrus orchard in Zhangzhou, Fujian, China. Total DNA was isolated using Plant Genomic DNA Kit (Tiangen Biotech Ltd., Beijing, China) and stored at the Fujian Agriculture and Forestry University (No. FAFUGX01). The strategy for sequencing, assembly, and annotating the chloroplast genome was adapted from Chen et al. (2019). The sequence of cp genomes was deposited in the GenBank (MN782007).

The whole complete cp genome of ‘Guanximiyou’ pummelo was a circle with 160,186 bp in size, containing a large single-copy region (LSC) of 87,939 bp, a small single-copy region (SSC) of 18,395 bp, and a pair of inverted repeat regions (IRA and IRB) of 26,926 bp. The cp DNA of ‘Guanximiyou’ pummelo comprised 109 unique genes, including 79 protein-coding genes (PCG), 29 tRNA genes, and 4 rRNA genes. Most of them (90) occur as a single copy, but 10 protein-coding genes (i.e. *ycf*1, *ycf*2, *ycf*15, *rps*7, *rps*12, *rps*19, *rpl*2, *rpl*22, *rpl*23, and *ndh*B), 8 tRNA genes (i.e. *trn*A-UGC, *trn*G-GCC, *trn*I-CAU, *trn*I-GAU, *trn*L-CAA, *trn*N-GUU, *trn*R-ACG, and *trn*V-GAC), and all the 4 rRNA genes (4.5S, 5S, 16S, and 23S rRNA) occur in double copies. The overall nucleation composition of the cp genome is 30.50% A, 31.10% T, 19.60% C, and 18.90% G, with the total GC content being 38.50%, while the corresponding value of the LSC, SSC, and IR region were 36.80, 33.30, and 43.00%, respectively.

To figure out its phylogenetic position, a ML tree was constructed based on published cp genome sequences of eight *Citrus* species, one *Atalantia*, one *Fortunella*, and one *Phellodendron* species using IQ tree (Nguyen et al. 2015) with 1000 bootstrap replicates. The 12 sequences were aligned using Mafft (Katoh and Standley 2013). The ModelFinder module in IQ tree was used to search the optimal nucleotide substitution model. Finally, the best model TIM + F+R3 was chosen to construct the phylogenetic tree according to BIC criteria using IQ tree. The result showed that ‘Guanximiyou’ pummelo is closely to *C. maxima*, *C. sinensis*, *C. platymamma*, and *C. limon* (Figure 1). The complete chloroplast genome of ‘Guanximiyou’ pummelo would provide valuable genetic information for its genetic relationships with other plant species of Rutaceae and would be subsequently used for citrus species researches.

**Figure 1.**
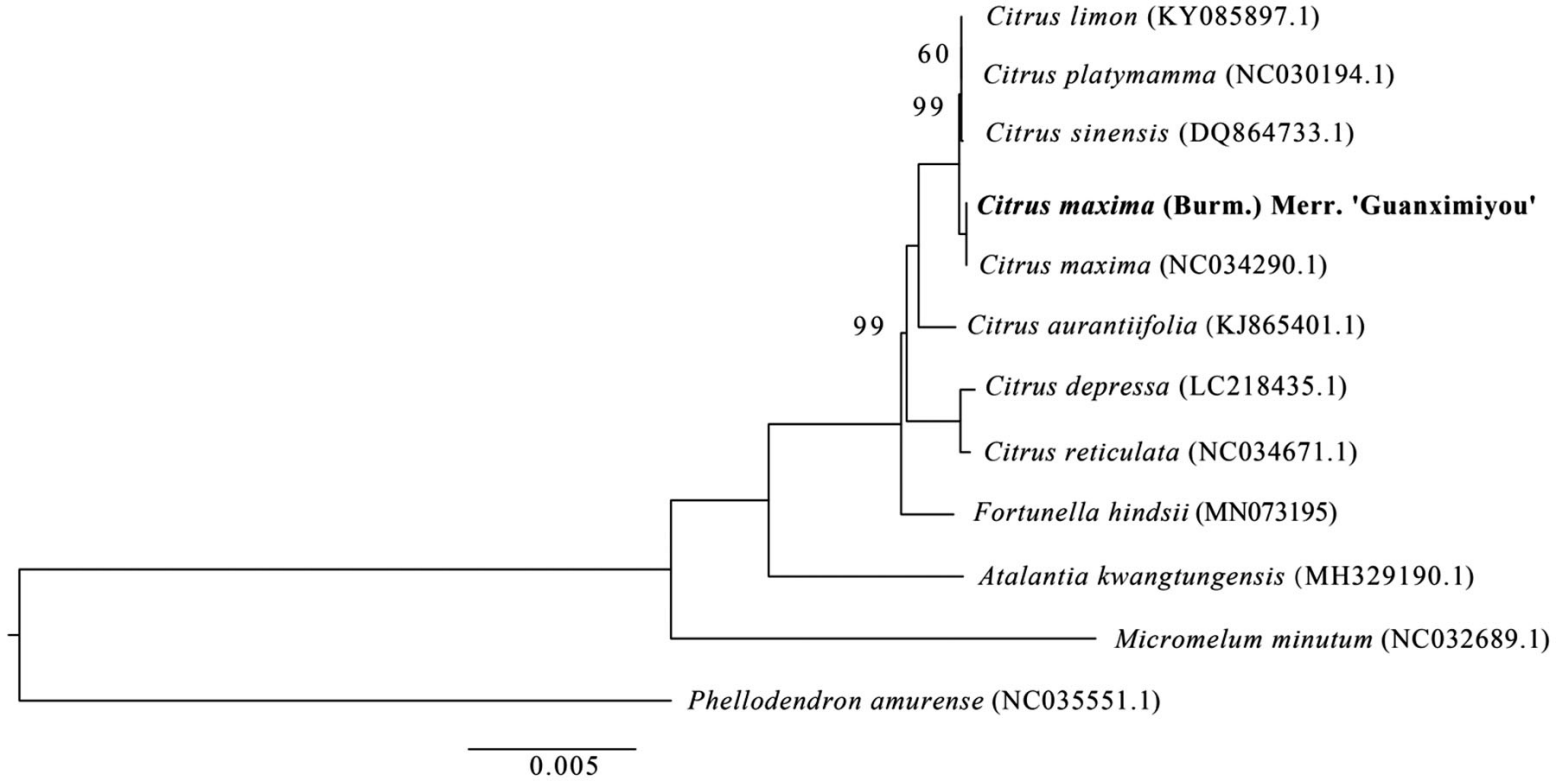
Maximum-likelihood tree based on the complete chloroplast genome sequences of ‘Guanximiyou’ pummelo and 11 plant species from the Rutaceae with *Phellodendron amurense* as outgroup. Numbers shown next to the nodes are bootstrap support values based on 1000 replicates, bootstrap values of 100 were omitted.
